# The role of AMP-activated protein kinase in the functional effects of vascular endothelial growth factor-A and -B in human aortic endothelial cells

**DOI:** 10.1186/2045-824X-3-9

**Published:** 2011-04-20

**Authors:** James A Reihill, Marie-Ann Ewart, Ian P Salt

**Affiliations:** 1 School of Pharmacy, Queens University Belfast, Lisburn Road, Belfast, UK; 2 Institute of Cardiovascular & Medical Sciences, College of Medicine, Veterinary & Life Sciences, University of Glasgow, Glasgow UK

## Abstract

**Background:**

Vascular endothelial growth factors (VEGFs) are key regulators of endothelial cell function and angiogenesis. We and others have previously demonstrated that VEGF-A stimulates AMP-activated protein kinase (AMPK) in cultured endothelial cells. Furthermore, AMPK has been reported to regulate VEGF-mediated angiogenesis. The role of AMPK in the function of VEGF-B remains undetermined, as does the role of AMPK in VEGF-stimulated endothelial cell proliferation, a critical process in angiogenesis.

**Methods:**

Human aortic endothelial cells (HAECs) were incubated with VEGF-A and VEGF-B prior to examination of HAEC AMPK activity, proliferation, migration, fatty acid oxidation and fatty acid transport. The role of AMPK in the functional effects of VEGF-A and/or VEGF-B was assessed after downregulation of AMPK activity with chemical inhibitors or infection with adenoviruses expressing a dominant negative mutant AMPK.

**Results:**

Incubation of HAECs with VEGF-B rapidly stimulated AMPK activity in a manner sensitive to an inhibitor of Ca^2+^/calmodulin-dependent kinase kinase (CaMKK), without increasing phosphorylation of endothelial NO synthase (eNOS) phosphorylation at Ser1177. Downregulation of AMPK abrogated HAEC proliferation in response to VEGF-A or VEGF-B. However, activation of AMPK by agents other than VEGF inhibited proliferation. Downregulation of AMPK abrogated VEGF-A-stimulated HAEC migration, whereas infection with adenoviruses expressing constitutively active mutant AMPK stimulated chemokinesis. Neither VEGF-A nor VEGF-B had any significant effect on HAEC fatty acid oxidation, yet prolonged incubation with VEGF-A stimulated fatty acid uptake in an AMPK-dependent manner. Inhibition of eNOS abrogated VEGF-mediated proliferation and migration, but was without effect on VEGF-stimulated fatty acid transport, ERK or Akt phosphorylation.

**Conclusions:**

These data suggest that VEGF-B stimulates AMPK by a CaMKK-dependent mechanism and stimulation of AMPK activity is required for proliferation in response to either VEGF-A or VEGF-B and migration in response to VEGF-A. AMPK activation alone was not sufficient, however, to stimulate proliferation in the absence of VEGF. VEGF-stimulated NO synthesis is required for the stimulation of proliferation by VEGF-A or VEGF-B, yet this may be independent of eNOS Ser1177 phosphorylation.

## Background

Vascular endothelial growth factor (VEGF)-mediated stimulation of endothelial cell proliferation and migration are key events in angiogenesis. Manipulation of VEGF signalling is seen as a promising therapeutic target for a number of disorders in which angiogenesis is inappropriate, yet the molecular mechanisms of action of VEGF in the endothelium are incompletely understood [1-3]. There are several members of the VEGF family expressed in humans. VEGF-A is thought to be the key VEGF family member that promotes angiogenesis, through the stimulation of endothelial cell proliferation, migration and survival [2,4-6]. VEGF-B is regarded as poorly angiogenic in most tissues except heart [6], yet improves survival in endothelial cells [7], such that its role in angiogenesis remains unclear. Recent studies have, however, identified a role for VEGF-B signalling in the regulation of fatty acid uptake in endothelial cells [8].

VEGFs bind to three related receptor tyrosine kinases, VEGF-R1, -R2 and -R3, with VEGF-R1 and VEGF-R2 largely restricted to endothelial cells [3]. VEGF-R2 binds VEGF-A, but not VEGF-B, and is considered to be the principal mediator of VEGF-A-regulated endothelial cell proliferation, angiogenesis and endothelial permeability [2,4,5]. The function of VEGF-R1, which binds both VEGF-A and VEGF-B, is less clear [3], but has been reported to promote endothelial cell survival [9], stimulate endothelial cell migration [10] and NO synthesis [11].

AMP-activated protein kinase (AMPK) is the downstream component of a protein kinase cascade that regulates cellular and whole body energy status [12]. Furthermore, it is now clear that AMPK is an important regulator of endothelial function [13]. We have demonstrated that VEGF-A stimulates AMPK activation, contributing in part to NO synthesis in cultured human aortic endothelial cells (HAECs) [14]. Furthermore, infection with adenoviruses expressing a dominant negative AMPK mutant inhibited VEGF-A-stimulated migration and endothelial tube formation under conditions of hypoxia in human umbilical vein endothelial cells (HUVECs), and reduced *in vivo *angiogenesis [15]. In addition, siRNA-mediated knockdown of AMPK has been reported to impair VEGF-A-stimulated bovine aortic endothelial cell (BAEC) migration and endothelial tube formation [16].

Taken together, these data indicate that AMPK is required for the angiogenic response to VEGF-A, yet whether AMPK mediates VEGF-B signalling has not been reported. Furthermore, although AMPK has been reported to regulate VEGF-mediated migration [16], the role of AMPK in endothelial cell proliferation, a key process in angiogenesis, remains uncharacterised. Finally, the role of AMPK in any VEGF-mediated alterations in fatty acid metabolism has similarly not been reported. In the current study, we examined the role of AMPK in the functional consequences of VEGF-A and VEGF-B signalling in HAECs, focussing on proliferation, migration and fatty acid metabolism.

## Methods

### Materials

Cryopreserved HAECs and cell culture media were obtained from PromoCell (Heidelberg, Germany). VEGF-B was from R&D systems (Abingdon, Oxfordshire, UK). A769662 was a generous gift from Prof. D. G. Hardie (University of Dundee, Dundee, UK). Compound C was obtained from Merck Chemicals Ltd. (Nottingham, UK). L-carnitine, palmitic acid, fatty acid-free bovine serum albumin, C_1_-BODIPY^®^500/510C_12_, Dowex 1X8-200 and VEGF_165 _(referred to as VEGF-A in this study) were from Sigma (Poole, Dorset, UK). [9,10(n)-^3^H]palmitic acid was from GE Healthcare Life Sciences (Little Chalfont, Buckinghamshire, UK). CellTiter AQueous one solution cell proliferation assay solution was obtained from Promega (Southampton, Hampshire, UK). All other reagents were from sources described previously [14,17,18].

### Cell culture

HAECs were grown in large vessel endothelial cell medium and used for experiments between passages 3 and 6 as described previously [14,17,18].

### Preparation of adenoviruses and infection of HAECs

Control adenoviruses (Ad.control) and adenoviruses expressing dominant negative mutant AMPKα1 (Ad.AMPK-DN) or constitutively active mutant AMPK (Ad.AMPK-CA) were propagated and purified as described previously [18]. HAECs were infected with 25 pfu/cell adenovirus in complete medium and the cells cultured for 48 h prior to experimentation.

### Preparation of cell lysates, SDS-PAGE and immunoblotting

Cells were incubated in Krebs Ringer Hepes (KRH) buffer (119 mM NaCl, 4.75 mM KCl, 1.2 mM MgSO_4_, 5 mM NaHCO_3_, 1.3 mM CaCl_2_, 20 mM Hepes-NaOH, pH 7.4, 5 mM glucose) for 3 h prior to the addition of test substances and further incubated for various durations at 37°C. The medium was removed; lysates (0.5 ml) prepared, resolved by SDS-PAGE and transferred to nitrocellulose. Nitrocellulose membranes were probed with the antibodies indicated as described previously [14,17,18].

### Immunoprecipitation and assay of AMPK

AMPK was immunoprecipitated from lysates and assayed using the SAMS substrate peptide as described previously [14,17]. Protein concentration was determined by the method of Bradford [19].

### HAEC proliferation assay

HAECs were cultured in complete media until 50-60% confluent in a 96-well plate and incubated in serum free culture medium for 2 h. Media was removed and replaced with basal endothelial cell culture medium supplemented with 0.2% (v/v) foetal calf serum. Cells were cultured for a further 20 h in the presence or absence of VEGF and/or inhibitors. HAEC proliferation was determined using the CellTiter 96^® ^AQueous One Solution Cell Proliferation solution according to the manufacturer's instructions. In experiments where AMPK adenovirus was used HAEC were infected for 24 h prior to serum starvation.

### HAEC migration assay

Migration assays were performed in a modified Boyden chamber using a 48-well chemotaxis chamber (Neuroprobe). A PCTE membrane was coated with 10 μg/ml type IV collagen in serum free medium supplemented with 0.5% (w/v) bovine serum albumin (BSA) for 1 h. The filter was placed over a bottom chamber containing VEGF in serum free medium-BSA. HAECs (1 × 10^6^cells/ml) were suspended in serum free medium-BSA and 50 μl added to each well in the upper chamber. HAECs were infected with adenovirus for 24 h before experimentation. The assembled chemotaxis chamber was incubated for 5 h and non-migrated cells on the upper surface of the filter were removed by scraping. The filter was stained with crystal violet and migrated cells quantified by counting the mean number of cells from 3 different fields at 40× magnification, performed in triplicate.

### Fatty acid oxidation assay

Palmitate oxidation was measured on the basis of ^3^H_2_O production. In order to account for intracellular fatty acid pools, HAECs cultured in 12 well plates until 90-95% confluent were pre-labelled with 2 μCi/ml [^3^H]-palmitic acid 24 h prior to the start of the experiment [20]. HAEC were incubated with 500 μl of 110 μM [^3^H]palmitic acid (8 μCi/ml), 50 μM carnitine, 0.5 mg/ml fatty acid-free BSA (FF-BSA) in Earle's-Hepes (116 mM NaCl, 5.3 mM KCl, 0.8 mM MgSO_4_, 1.8 mM CaCl_2_, 1 mM NaH_2_PO_4_, 20 mM Hepes-NaOH, pH 7.4) in the presence or absence of AICAR or VEGF for 4 h at 37°C. At the end of the incubation period ^3^H_2_O was separated from the unreacted substrate by the addition of 30 μl of 10% (w/v) FF- BSA. TCA (72% (w/v), 50 μl) was then added and incubated at 4°C for 10 min before centrifugation at 2000 × *g *for 10 min at 4°C. The supernatant was removed, the pH adjusted to 7-9 using 1 M NaOH and transferred to a Dowex 1X8-200 ion exchange column. Columns were eluted with 2 ml of deionised water and ^3^H_2_O assessed by liquid scintillation counting.

### Fatty acid uptake assay

Confluent HAECs were cultured in 12 well plates in serum-free growth medium supplemented with 1% (v/v) FF-BSA in the presence or absence of test substances for 24 h before being washed with PBS containing 1% (v/v) FF-BSA and incubated at 37°C for 3 min in PBS, 1% (v/v) FF-BSA, 110 μM [^3^H]-palmitic acid (6 μCi/ml). [^3^H]-palmitic acid transport was terminated by sequential washing in ice-cold PBS. Cells were lysed in 1% (v/v) Triton X-100 and cell-associated radioactivity assessed by scintillation counting.

### Statistics

Unless stated otherwise, results are expressed as the mean ± S.E. Statistically significant differences were determined using a 2-tailed Student's *t *test, with *p *< 0.05 as significant.

## Results

### VEGF-B stimulates AMPK via CaMKK in HAECs

**Figure 1:**
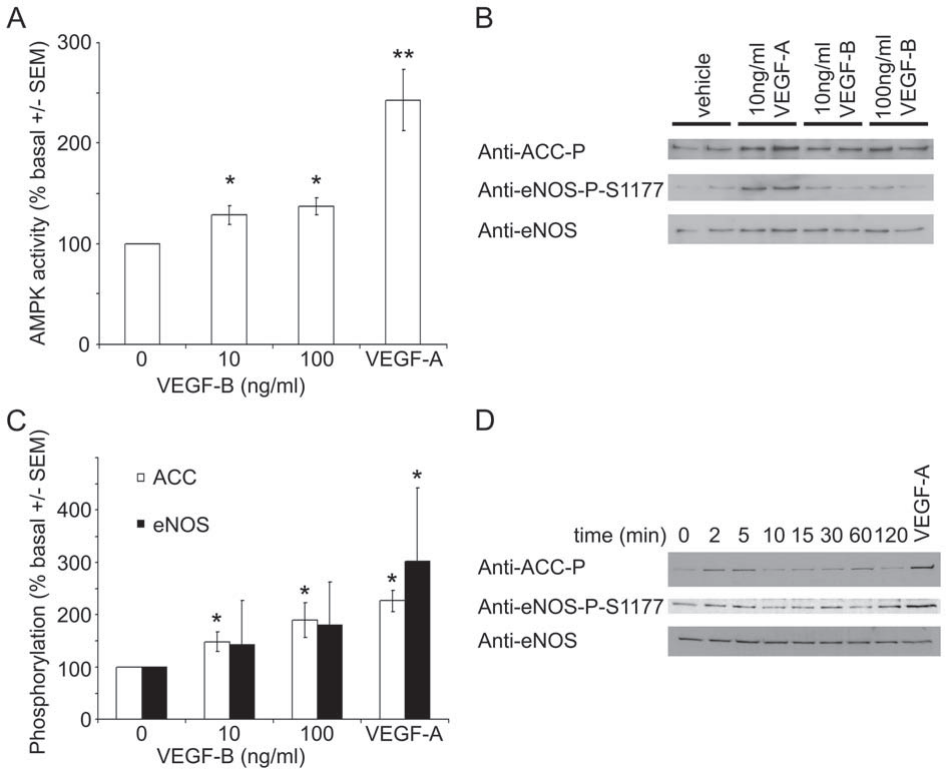
**VEGF-B stimulates AMPK in HAECs**. HAECs were incubated in the presence or absence of A-C) the indicated concentrations of VEGF-A or VEGF-B for 5 min or D) 100 ng/ml VEGF-B for the durations indicated and lysates prepared. Lysates were subsequently subjected to A) assay of AMPK activity or B,C,D) immunoblotting with the antibodies indicated. A) The results are expressed as the mean ± SEM% basal AMPK activity for four independent experiments. B,D) Representative immunoblots are shown, repeated with similar results on three independent sets of lysates. C) Quantification of immunoblots. *p < 0.05 relative to value in absence of VEGF, **p < 0.01 relative to value in absence of VEGF.

**Figure 2:**
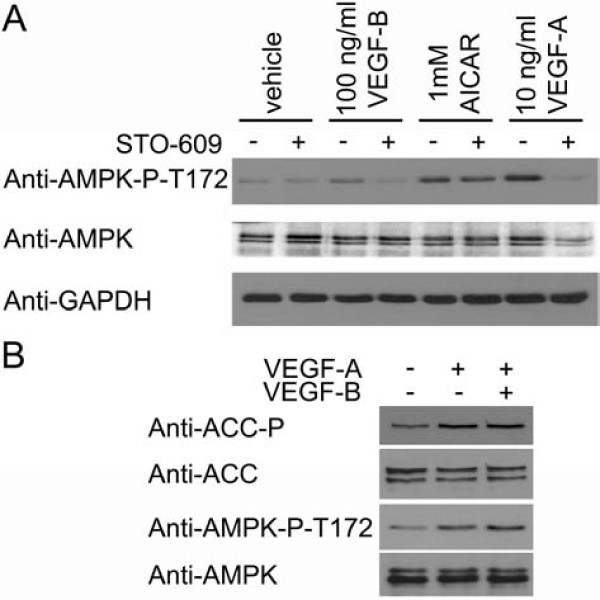
**VEGF-B-stimulated AMPK Thr172 phosphorylation is inhibited by STO-609**. A) HAECs were preincubated in the presence or absence of 10 μM STO-609 for 15 min prior to incubation with the indicated concentrations of AICAR for 45 min or VEGF-A and VEGF-B for 5 min and lysates prepared. B) HAECs were incubated with VEGF-A (10 ng/ml) and/or VEGF-B (100 ng/ml) for 5 min and lysates prepared. Lysates were subsequently subjected to immunoblotting with the antibodies indicated. Representative immunoblots are shown, repeated with similar results on three independent sets of lysates.

Incubation of HAECs for 5 minutes with 10 or 100 ng/ml VEGF-B significantly stimulated AMPK activity, yet the magnitude of this stimulation was less than that elicited by 10 ng/ml VEGF-A (Figure 1A). VEGF-B also significantly stimulated phosphorylation of the AMPK substrate, acetyl CoA carboxylase (ACC) (Figure 1B &1C), which reached a maximum after 2-5 min before returning to basal levels of phosphorylation (Figure 1D). Unlike VEGF-A, VEGF-B had no significant effect on eNOS Ser1177 phosphorylation in HAECs (Figure 1B-D). We have previously reported that VEGF-A stimulates AMPK by a CaMKK-mediated mechanism [14]. We therefore determined whether the stimulation of AMPK by VEGF-B was sensitive to the CaMKK inhibitor, STO-609. Preincubation of HAECs with STO-609 completely inhibited VEGF-B and VEGF-A-stimulated AMPK phosphorylation at Thr172, yet had no effect on AMPK Thr172 phosphorylation stimulated by AICAR (Figure 2A), which stimulates AMPK phosphorylation by a CaMKK-independent mechanism [12-14,17]. Incubation of HAECs with VEGF-B had no additional effect on VEGF-A-stimulated AMPK Thr172 or ACC phosphorylation (Figure 2B).

### VEGF-stimulated HAEC proliferation requires AMPK activity

**Figure 3:**
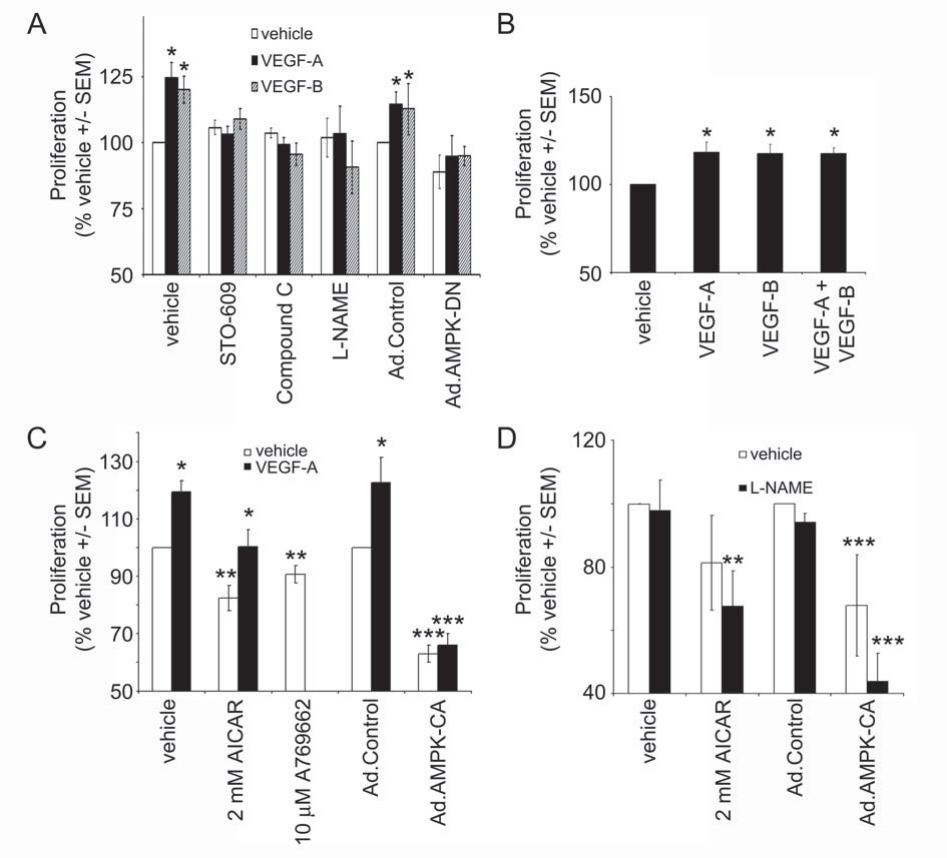
**AMPK is required for VEGF-stimulated HAEC proliferation**. HAECs were incubated in the presence or absence of VEGF-A (10 ng/ml), VEGF-B (100 ng/ml), Compound C (5 μM), STO-609 (5 μM), AICAR (2 mM), A769662 (10 μM), L-NAME (1 mM) for 24 h and proliferation assessed. Ad.control, Ad.AMPK-DN and Ad.AMPK-CA adenoviruses were used to infect HAECs 24 h prior to the addition of VEGF. Results are expressed as the mean ± SEM basal proliferation for three independent experiments or six independent experiments in the case of Ad.AMPK-DN. *p < 0.05 relative to absence of VEGF, **p < 0.05 relative to absence of AICAR or A769662, ***p < 0.05 relative to Ad.control.

We next determined the role of AMPK in VEGF-stimulated HAEC proliferation. Both VEGF-A and VEGF-B significantly stimulated HAEC proliferation in cells cultured in low serum (0.2% (v/v)) concentrations. Incubation with the AMPK inhibitor, compound C, or STO-609 prevented the stimulation of proliferation in response to either VEGF-A or VEGF-B. Furthermore, incubation with the eNOS inhibitor, L-NAME also completely abrogated VEGF-A and VEGF-B-stimulated HAEC proliferation (Figure 3A). Co-incubation of HAECs with VEGF-A and VEGF-B elicited no significant increase in HAEC proliferation when compared to stimulation with either VEGF isoform alone (Figure 3B). Neither VEGF-A nor VEGF-B were able to elicit proliferation in HAECs infected with Ad.AMPK-DN, whereas either VEGF-A or VEGF-B stimulated proliferation in Ad.control-infected HAECs (Figure 3A). In contrast, AMPK activation in HAECs with AICAR, A769662 or infection of HAECs with Ad.AMPK-CA all reduced basal cell proliferation (Figure 3C). VEGF-A was able to stimulate proliferation in cells coincubated with AICAR, yet had no effect in cells overexpressing constitutively active AMPK (Figure 3C). Preincubation with L-NAME had no significant effect on the inhibition of HAEC proliferation in response to AICAR or infection with Ad.AMPK-CA (Figure 3D).

### AMPK does not regulate VEGF-stimulated ERK1/2 or Akt phosphorylation

**Figure 4:**
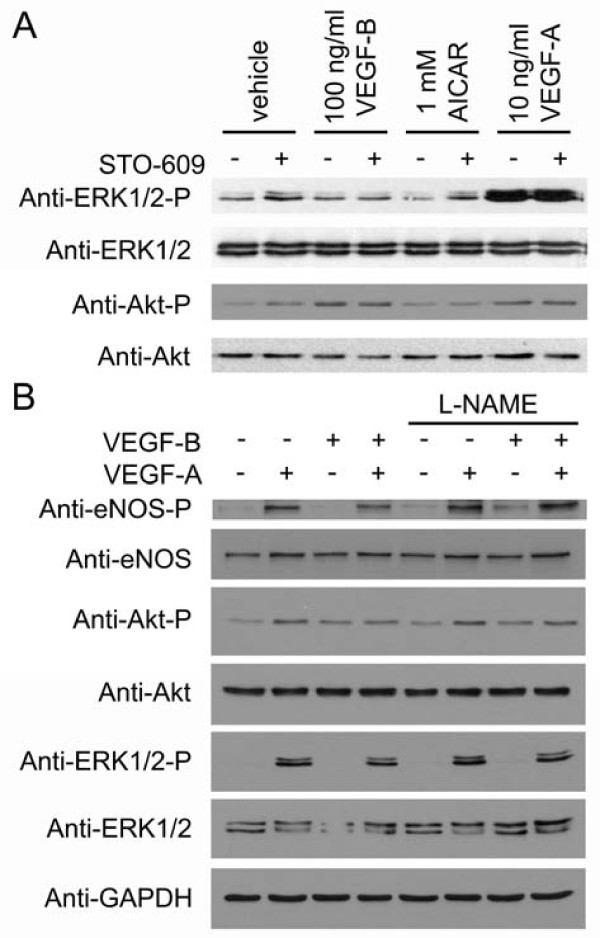
**Inhibition of AMPK or eNOS has no effect on VEGF-stimulated ERK1/2 or Akt phosphorylation**. HAECs were preincubated in the presence or absence of A) 10 μM STO-609 or B) 1 mM L-NAME for 15 min prior to incubation with the indicated concentrations of AICAR for 45 min or VEGF-A and VEGF-B for 5 min and lysates prepared. Lysates were subsequently subjected to immunoblotting with the antibodies indicated. Representative immunoblots are shown, repeated with similar results on three independent sets of lysates.

The activities of ERK and Akt have been demonstrated to be required for endothelial cell proliferation and survival respectively [4]. We therefore determined whether ERK or Akt phosphorylation was altered by AMPK activity. VEGF-A caused a robust increase in ERK phosphorylation, whereas neither VEGF-B, nor AICAR altered ERK phosphorylation (Figure 4A). Akt phosphorylation was stimulated by VEGF-A and VEGF-B, but unaltered by AICAR (Figure 4A). Furthermore; STO-609 had no effect on basal or stimulated ERK or Akt phosphorylation (Figure 4A). Co-stimulation with VEGF-B did not alter VEGF-A-stimulated Akt, ERK or eNOS phosphorylation, and preincubation with L-NAME had no effect on either VEGF-A-stimulated ERK, eNOS or Akt phosphorylation or VEGF-B-stimulated Akt phosphorylation (Figure 4B)

### VEGF-A stimulates HAEC migration in an AMPK-dependent manner

**Figure 5:**
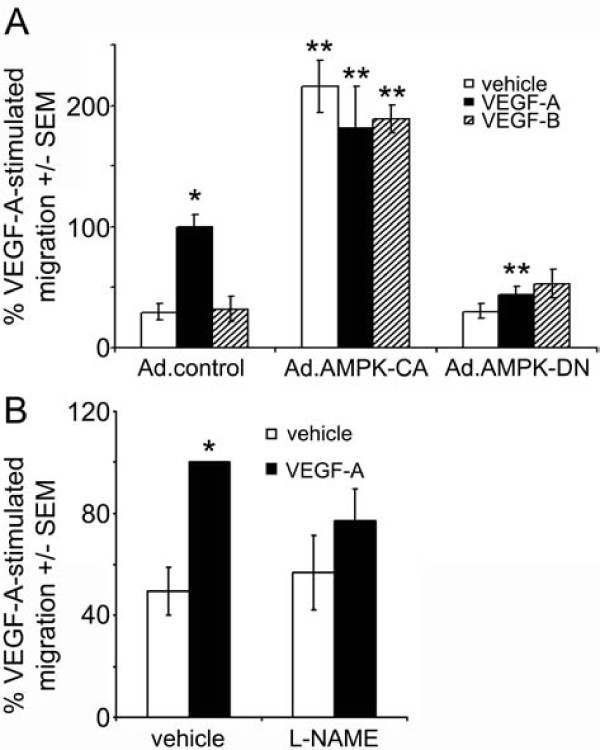
**AMPK is required for VEGF-A-stimulated migration**. A) HAECs were infected with Ad.control, Ad.AMPK-DN or Ad.AMPK-CA for 24 h prior to assessment of migration in response to 10 ng/ml VEGF-A or 100 ng/ml VEGF-B using a Boyden chamber. B) HAECs were incubated in the presence or absence of L-NAME (1 mM) 60 min prior to incubation with VEGF-A (10 ng/ml) for 24 h and migration assessed. The results are expressed as the mean ± SEM% VEGF-stimulated migration for three independent experiments. *p < 0.001 relative to absence of VEGF, ** p < 0.001 relative to Ad.control-infected cells.

VEGF-A markedly stimulated HAEC migration, whereas VEGF-B had no significant effect on HAEC migration (Figure 5A). Infection of HAECs with Ad.AMPK-DN prevented VEGF-A-stimulated HAEC migration, while infection with Ad.AMPK-CA stimulated migration in the absence or presence of VEGF-A or VEGF-B (Figure 5A). Preincubation with L-NAME attenuated VEGF-A-stimulated HAEC migration (Figure 5B)

### VEGF has no effect on fatty acid oxidation in HAECs

**Figure 6:**
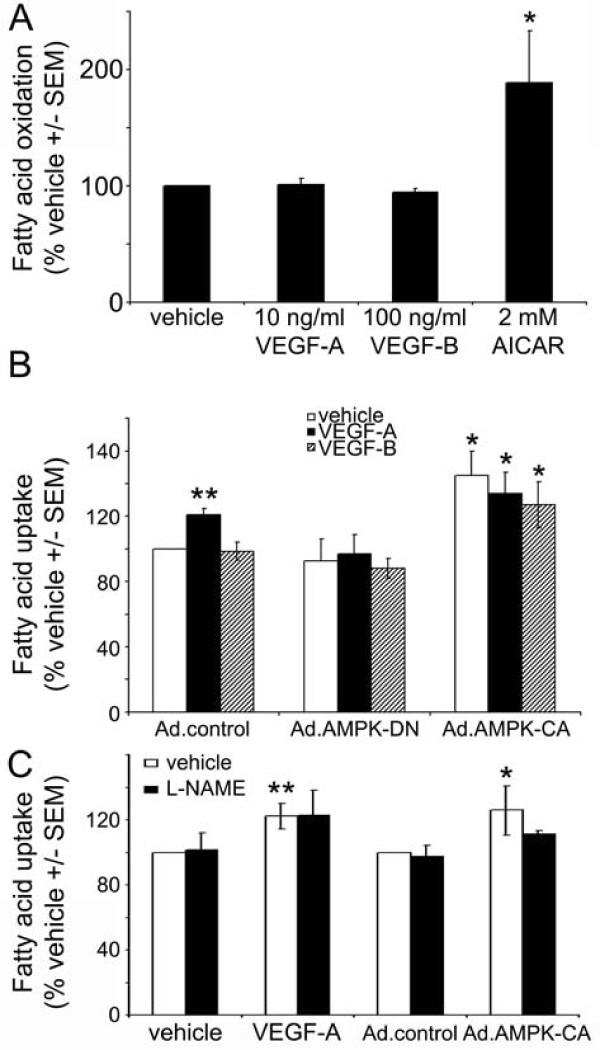
**The effect of VEGF on fatty acid oxidation and uptake**. A) Fatty acid oxidation was assessed in HAECs incubated in the presence or absence of the indicated concentrations of VEGF-A, VEGF-B or AICAR for 4 h. B) HAECs were incubated in the presence or absence of VEGF-A (25 ng/ml) or VEGF-B (250 ng/ml) for 24 h and fatty acid uptake assessed. Ad.control, Ad.AMPK-DN and Ad.AMPK-CA adenoviruses were used to infect HAECs 24 h prior to the addition of VEGF. C) HAECs were incubated in the presence or absence of VEGF-A (25 ng/ml) and L-NAME (1 mM) for 24 h and fatty acid uptake assessed. Results are expressed as A) the mean ± SEM basal fatty acid oxidation or B,C) the mean ± SEM basal fatty acid uptake for 3 independent experiments performed in triplicate. *p < 0.05 relative to Ad.control-infected HAECs. **p < 0.05 relative to vehicle.

ACC phosphorylation by AMPK inhibits ACC activity, reducing malonyl CoA synthesis, leading to increased fatty acid oxidation in several tissues [12]. Indeed, incubation of HUVECs with AICAR has previously been demonstrated to stimulate fatty acid oxidation [20]. As VEGF stimulated ACC phosphorylation (Figure 1), we determined the effect of VEGF-A and VEGF-B on fatty acid oxidation. Stimulation of HAECs with AICAR was demonstrated to stimulate fatty acid oxidation 1.9 ± 0.4-fold, yet neither VEGF-A nor VEGF-B had any effect on HAEC fatty acid oxidation (Figure 6A).

### VEGF-A stimulates HAEC fatty acid uptake in an AMPK-dependent manner

Recent studies have reported that prolonged incubation with VEGF-B stimulates fatty acid uptake in several endothelial cell types [8]. We next determined whether VEGF-A or VEGF-B stimulated fatty acid uptake in HAECs. Incubation of confluent HAECs for 24 h with VEGF-A stimulated the uptake of [^3^H]-palmitic acid, yet VEGF-B had no effect on the uptake of [^3^H]-palmitic acid (Figure 6B). Stimulation of fatty acid uptake by VEGF-A was no longer apparent in HAECs infected with Ad.AMPK-DN compared to Ad.control-infected cells (Figure 6B). Infection with Ad.AMPK-CA stimulated basal fatty acid uptake, yet incubation with VEGF-A or VEGF-B had no further effect on fatty acid uptake in Ad.AMPK-CA infected cells (Figure 6B). Incubation of HAECs with L-NAME had no effect on VEGF-A-stimulated fatty acid uptake, whereas although L-NAME appeared to suppress Ad.AMPK-CA-stimulated fatty acid uptake, this did not reach statistical significance (p = 0.19).

## Discussion

The principal findings of this study are that i) VEGF-B stimulates AMPK activity in HAECs and ii) AMPK is required for HAEC proliferation in response to either VEGF-A or VEGF-B. Incubation of HAECs with VEGF-B robustly stimulated AMPK activity, yet the extent of activation was modest when compared with VEGF-A. There was no additive effect of VEGF-B on VEGF-A-stimulated AMPK activity. VEGF-B activates VEGF-R1, whereas VEGF-A activates both VEGF-R1 and VEGF-R2. These findings suggest that VEGF-R1 stimulation leads to AMPK activation. Stimulation of AMPK by VEGF-B was sensitive to the CaMKK inhibitor, STO-609, as we have previously demonstrated for VEGF-A [14], implying that VEGF-B, via VEGF-R1 stimulates an increase in intracellular Ca^2+ ^required for activation of CaMKK. One previous study using BAECs has reported that siRNA-mediated downregulation of VEGF-R2 abrogated phosphorylation of AMPK and ACC in response to VEGF, although the particular VEGF used was not explicitly stated [16]. When these data are taken together with those in the current study, it suggests that stimulation of VEGF-R1 or VEGF-R2 activates AMPK via a CaMKK-mediated mechanism in HAECs.

Angiogenesis involves endothelial cell proliferation as well as the migration of the cells toward an angiogenic stimulus. Although VEGF-A is a well-documented stimulus for proliferation and migration [2,4-6], there are few published studies of the effects of VEGF-B on endothelial cell proliferation, survival or migration. VEGF-B has been reported to stimulate proliferation in bovine carotid artery endothelial cells [21] and increase survival of mouse retinal or choroidal endothelial cells [7]. The stimulation of HAEC proliferation in response to VEGF-B observed in the current study represents, to the authors' knowledge, the first demonstration that VEGF-B stimulates proliferation in isolated human endothelial cells. HAEC proliferation in response to either VEGF-A or VEGF-B was abrogated by compound C, STO-609 or infection with Ad.AMPK-DN. These observations establish AMPK as a key regulator of cell proliferation in response to VEGF in HAECs. Such findings are consistent with previous observations demonstrating AMPK-mediated angiogenesis in response to both adiponectin [22] and hypoxia [15].

Despite the requirement for AMPK in VEGF-stimulated endothelial cell proliferation, activation of AMPK with AICAR, A769662 or Ad.AMPK-CA suppressed proliferation in the absence of VEGF. This effect of AMPK activation alone is consistent with studies in non-endothelial cells, where AMPK activation has been reported to cause cell cycle arrest [23-25] and to facilitate apoptosis [26]. We propose that although AMPK activation is required for VEGF-stimulated proliferation, activation of AMPK in the absence of VEGF suppresses proliferation. In agreement with this hypothesis, VEGF was able to stimulate proliferation in AICAR-treated cells. In contrast, however, VEGF was unable to stimulate proliferation in cells infected with Ad.AMPK-CA. As the HAECs were infected with Ad.AMPK-CA 24 h prior to the addition of VEGF, the inability of VEGF to stimulate proliferation may reflect an inability of VEGF to overcome sustained prior inhibition of proliferation.

VEGF stimulates a variety of signalling pathways which are, in addition to AMPK, reported to be necessary for a proliferative response. VEGF-stimulated ERK activation has similarly been reported to be necessary for VEGF-induced proliferation, but is not sufficient to induce proliferation when it occurs in the absence of VEGF [27,28]. VEGF-R2-mediated stimulation of Akt has been reported to be required for the survival of endothelial cells [4,29]. Activation of AMPK with AICAR or inhibition of VEGF-stimulated AMPK activity with STO-609 had no effect on basal or VEGF-stimulated ERK or Akt phosphorylation, indicating that these kinases are not downstream of AMPK activation. Therefore, AMPK may act in concert with Ca^2+^-independent pathways such as ERK and Akt activation to regulate proliferation. An alternative possible cause for the opposing effects of AMPK activation in the presence or absence of VEGF is that the AMPK activated by VEGF localizes to different regions of the cell than that activated by AICAR or A769662.

NO is a key regulator of VEGF-stimulated proliferation [30], and we have previously demonstrated VEGF-stimulated NO synthesis is, in part, downstream of AMPK activation in HAECs [14] and therefore may be responsible for the proliferative effects mediated by AMPK in HAECs. Indeed, inhibition of eNOS with L-NAME suppressed HAEC proliferation in response to VEGF-A or VEGF-B, in agreement with previous studies using VEGF-A [30]. Intriguingly, however, VEGF-B stimulated proliferation and AMPK activity without significantly altering phosphorylation of eNOS at Ser1177, indicating that, in the case of VEGF-B, direct phosphorylation of eNOS at Ser1177 is not required for the stimulation of proliferation. Phosphorylation of eNOS at Ser1177 is not the only mechanism by which VEGF acts to stimulate nitric oxide synthesis, as VEGF has been demonstrated to stimulate PLCγ-mediated increases in intracellular Ca^2+ ^[30,31], which act to directly stimulate eNOS independent of phosphorylation. Indeed, it has recently been reported that VEGF-A-stimulated phosphorylation of eNOS at Ser1177 was unaffected by downregulation of AMPK by specific siRNA, yet VEGF-stimulated angiogenesis was impaired in AMPK knockout animals [32]. It remains possible, therefore, that VEGF-B and VEGF-A stimulate NO synthesis independently of either eNOS phosphorylation at Ser1177 or AMPK and that VEGF-stimulated NO synthesis and AMPK activation are required for HAEC stimulated proliferation. Furthermore, L-NAME had no effect on VEGF-stimulated ERK or Akt phosphorylation, indicating that stimulation of these pathways is independent of VEGF-stimulated NO synthesis.

In the current study, VEGF-A, and not VEGF-B stimulated migration of HAECs. Downregulation of AMPK with adenoviruses expressing dominant negative mutant AMPK or inhibition of eNOS with L-NAME completely abrogated the stimulation by VEGF-A. This is in agreement with a previous study, in which VEGF-stimulated migration of BAECs was shown to be attenuated by downregulation of AMPK or eNOS using siRNA [16]. Similarly, infection of HUVECs with adenoviruses expressing dominant negative mutant AMPKα2 has been reported to ablate VEGF-stimulated migration in HUVECs under hypoxic conditions [15]. The lack of effect of VEGF-B on HAEC migration may reflect the relatively modest stimulation of AMPK (when compared to VEGF-A or Ad.AMPK-CA). Alternatively, it is possible that VEGF-B does not stimulate AMPK activity in the correct intracellular compartment to facilitate migration.

For the first time, we also demonstrated that infection of HAECs with Ad.AMPK-CA resulted in a significant increase in cell migration in the absence of VEGF, indicative of increased random cell movement (chemokinesis). Such an increase in chemokinesis may be a mechanism by which endothelial cells seek nutrients when their energy status is compromised with resultant AMPK activation. The AMPK activators, AICAR and phenformin have been reported to inhibit chemokinesis in U937 promonocytic cells [33]. The different effects of AMPK observed in the two contrasting studies are likely to reflect the different cell types used and the potential pleitropic effects of AICAR and phenformin.

A number of reports have demonstrated that AMPK activation in response to AICAR, glucose deprivation and bradykinin stimulates fatty acid oxidation in endothelial cells [20,34]. However, in the present study neither VEGF-A nor VEGF-B had any effect on fatty acid oxidation in HAECs, indicating that the transient phosphorylation of ACC in response to VEGF, unlike the more prolonged effect of AICAR, may not be sufficient to significantly increase fatty acid oxidation.

It has recently been reported that VEGF-B signalling upregulates fatty acid uptake in endothelial cells, a possible consequence of increased expression of fatty acid transport proteins (FATPs) [8]. Intriguingly, in confluent HAECs, stimulation for 24 h with VEGF-A, but not VEGF-B caused a modest, yet significant stimulation of fatty acid transport. This effect was abrogated by infection of HAECs with Ad.AMPK-DN and mimicked by infection with Ad.AMPK-CA. There was no significant effect of L-NAME on VEGF-A-stimulated fatty acid uptake. These data indicate that, in HAECs, VEGF-A stimulates fatty acid transport in an AMPK-dependent, NO-independent manner. The lack of effect of VEGF-B with respect to fatty acid transport may reflect the different source of endothelial cells (HAECs) used in the current study.

## Conclusions

We propose that AMPK activation is required for endothelial cell proliferation stimulated by VEGF-A or VEGF-B, and migration stimulated by VEGF-A, two mechanisms that underlie the regulation of angiogenesis by AMPK. Conversely, activation of AMPK in the absence of VEGF, via the different signalling pathways utilized by AICAR or A769662 suppresses proliferation and stimulates chemokinesis. VEGF-stimulated NO synthesis is required for proliferation and migration, yet may not be dependent on VEGF-stimulated eNOS phosphorylation. Inhibition of cell proliferation by AMPK in response to reduced energy status, in the absence of the distinct signalling pathways induced by angiogenic factors such as VEGF, represents a facet of the classical energy-conserving role of AMPK.

## Competing interests

The authors declare that they have no competing interests.

## Authors' contributions

JAR carried out the AMPK assays, immunoblotting, fatty acid oxidation and some of the proliferation assays. MAE carried out the migration assays and some of the proliferation assays. The study was conceived by IPS, who contributed to data collection and wrote the manuscript. All authors read and approved the final manuscript.
